# Virginia

**Published:** 1897-04

**Authors:** 


*Virginia.* The subject of meat- and dairy-inspection is being agitated in some of the cities of the State. The Town Council of Blacksburg passed an ordinance a short time ago establishing meat- and dairy-inspection for the town; also establishing a board of health, consisting of two physicians and one veterinarian.

The University of Virginia has requested the State Veterinarian to make the tuberculin-test on all dairy herds supplying milk to the University boarding-hall.

As a result of the work done on Texas cattle-fever in Virginia in 1896 over six counties have this year been released from quarantine. The State is making a strong effort to stamp out the disease.

